# An optimal window of platelet reactivity by LTA assay for patients undergoing percutaneous coronary intervention

**DOI:** 10.1186/s12959-021-00323-5

**Published:** 2021-10-19

**Authors:** Jing Wang, Jing Wang, Zhou Dong, Jiazheng Ma, Jianzhen Teng, Tong Wang, Xiaofeng Zhang, Qian Gu, Zekang Ye, Inam Ullah, Chuchu Tan, Samee Abdus, Lu Shi, Xiaoxuan Gong, Chunjian Li

**Affiliations:** 1 Departments of Cardiology, the First Affiliated Hospital of Nanjing Medical University, 300 Guangzhou Road, Nanjing, 210029 Jiangsu China; 2 Department of Cardiology, the Affiliated Huaian No.1 People’s Hospital of Nanjing Medical University, Huai’an, Jiangsu China; 3 Department of Cardiology, the First People’s Hospital of Yancheng, Yancheng, Jiangsu China; 4 Department of Cardiology, the Second Hospital of Nanjing, Nanjing University of Chinese Medicine, Nanjing, Jiangsu China

**Keywords:** Light transmittance aggregometry, Platelet reactivity, Percutaneous coronary intervention, Therapeutic window

## Abstract

**Objective:**

This study was aimed to determine how platelet reactivity (PR) on dual antiplatelet therapy predicts ischemic and bleeding events in patients underwent percutaneous coronary intervention (PCI).

**Design:**

A total of 2768 patients who had received coronary stent implantation and had taken aspirin 100 mg in combination with clopidogrel 75 mg daily for > 5 days were consecutively screened and 1885 were enrolled. The recruited patients were followed-up for 12 months. The primary end-point was the net adverse clinical events (NACE) of cardiovascular death, nonfatal myocardial infarction (MI), target vessel revascularization (TVR), stent thrombosis (ST) and any bleeding.

**Result:**

1709 patients completed the clinical follow-up. By using the receiver operating characteristic (ROC) curve analysis, the optimal cut-off values were found to be 37.5 and 25.5% respectively in predicting ischemic and bleeding events. Patients were classified into 2 groups according to PR: inside the window group (IW) [adenosine diphosphate (ADP) induced platelet aggregation (PL_ADP_) 25.5–37.4%)] and outside the window group (OW) (PL_ADP_ < 25.5% or ≥ 37.5%). The incidence of NACE was 16.8 and 23.1% respectively in the IW and OW group. The hazard ratio of NACE in IW group was significantly lower [0.69 (95% CI, 0.54–0.89, *P* = 0.004)] than that in the OW group during 12-month follow-up.

**Conclusion:**

An optimal therapeutic window of 25.5–37.4% for PL_ADP_ predicts the lowest risk of NACE, which could be referred for tailored antiplatelet treatment while using LTA assay.

**Trial registration:**

Trial registration number: ClinicalTrials.govNCT01968499. Registered 18 October 2013 - Retrospectively registered.

## Introduction

Dual antiplatelet therapy with aspirin and an adenosine diphosphate (ADP)-receptor (P2Y_12_) inhibitor is a cornerstone of the pharmacological treatment for patients with coronary artery disease undergoing percutaneous coronary intervention (PCI) [1].

Clopidogrel is one of the most widely used P2Y_12_ inhibitors, which undergoes a two-step metabolic transformation before binding to the platelet P2Y_12_ receptor [2]. Studies have shown wide variability of platelet clopidogrel response [3], indicating that a substantial proportion of patients have inappropriate platelet inhibition at a regular dose of clopidogrel 75 mg once daily. It has been reported that high on-treatment platelet reactivity (HOPR) detected by platelet aggregometry leads to increased risk of thrombotic events [4–8], while low on-treatment platelet reactivity (LOPR) leads to increased risk of bleeding after PCI [9, 10]. Thus, it is important to identify an optimal platelet inhibition or on-treatment platelet reactivity (PR) by platelet aggregometry [11, 12].

This study was to investigate an optimal therapeutic window for PR determined by light transmission aggregometry (LTA) to predict the lowest ischemic and bleeding risks in patients underwent PCI and treated with dual antiplatelet agents.

## Methods

This is a prospective, single-center, registration study conducted at the First Affiliated Hospital of Nanjing Medical University, Nanjing, China. The study was registered at URL: https://www.clinicaltrials.gov (Unique identifier: NCT01968499) and was approved by the ethics committee of the First Affiliated Hospital of Nanjing Medical University. Written informed consent was obtained from each patient.

### Study population

**Fig. 1:**
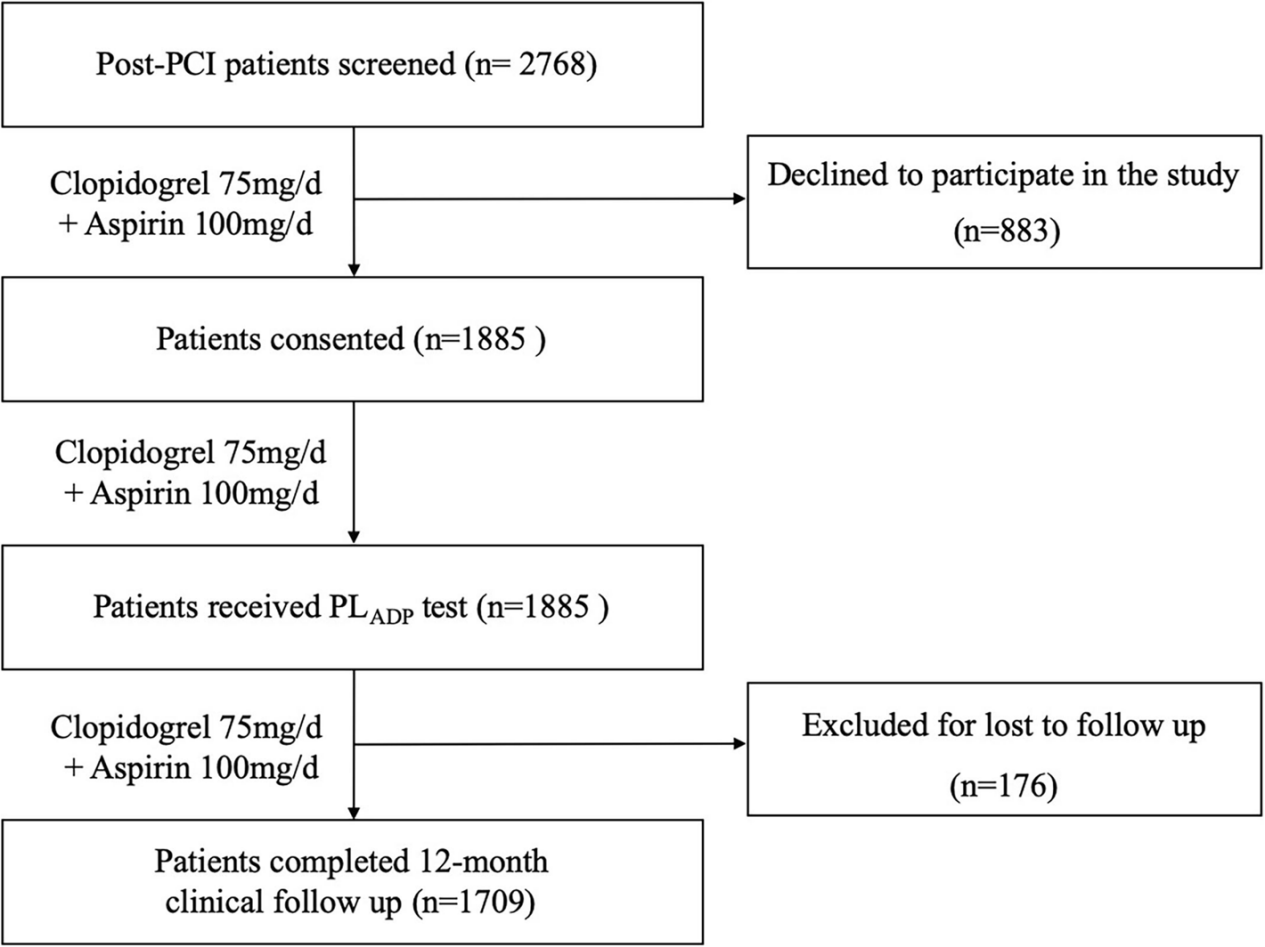
Study Flow Chart

A total of 2768 patients were consecutively screened from April 2011 to October 2016 in the First Affiliated Hospital of Nanjing Medical University, among which 883 declined to participate, and the remaining 1885 patients were enrolled in the study (Fig. 1).

The inclusion criteria were patients who had undergone coronary stent implantation and taken aspirin 100 mg in combination with clopidogrel 75 mg daily for > 5 days [7]. Exclusion criteria were patients: 1) intolerant to aspirin or clopidogrel (e.g. history of allergic reactions or gastrointestinal bleeding); 2) taking any other antiplatelet agents in addition to aspirin and clopidogrel (e.g. cilostazol); 3) taking any anticoagulant agents (e.g. vitamin K antagonists, new oral anticoagulants); 4) with myelodysplastic syndrome or abnormal baseline platelet counts of < 80 × 10^9^/L or > 450 × 10^9^/L; 5) with hemoglobin < 90 g/L; 6) with cancer or any other complications that may not suitable to be recruited at the discretion of the investigators.

### PR measurements

Six milliliter venous blood was collected into 3.2% citrate vacutainer tubes in the morning 2 h after the patients’ taking clopidogrel (if glycoprotein (GP) IIb/IIIa inhibitors were used, testing would be performed 24 h after drug discontinuation). Blood samples were subjected to platelet function test by LTA within 2 h as previously described [13]. In brief, samples were centrifuged at 200 g for 8 min to obtain platelet-rich plasma (PRP). Platelet-poor plasma (PPP) was prepared by centrifuging the remaining blood at 2465 g for 10 min. Platelet counts were adjusted by the addition of PPP to the PRP to achieve a count of 250 × 10^9^/L. The ADP-induced platelet aggregation (PL_ADP_) was recorded using the maximum platelet aggregation within 8 min after addition of ADP (final concentration 5 μmol/L) by a Chronolog Model 700 aggregometer (Chrono-log Corporation, Havertown, PA, USA) [13].

### Study end-points

The primary end-point was set as the net adverse clinical events (NACE), a composite of ischemic events including cardiovascular death, nonfatal myocardial infarction (MI), target vessel revascularization (TVR), stent thrombosis (ST) and any bleeding defined by the Thrombolysis in Myocardial Infarction (TIMI) criteria [14]. MI was defined in accordance with the Third Universal Definition proposed in 2007 [15]. ST was defined as definite or probable according to the Academic Research Consortium definitions [16]. All the clinical events were independently adjudicated by two investigators blinded to the results of PR tests. Disagreements were resolved by discussion or consultation with a third investigator (Li).

The outcome data were collected by 2 investigators who were blinded to the results of platelet reactivity testing. The patients were followed up in the clinic and less preferably by telephone call if they were unable to attend the clinic. A standard case report form was used to record the outcome.

### Statistical analysis

Statistical analysis was performed using SPSS 22.0 soft-ware (SPSS, Chicago, IL, USA). Continuous variables are expressed as means ± standard deviations (SD) or medians (range [or Inter Quartile Range]). Categorical variables are expressed as frequencies and percentages. Two-sided Mann–Whitney tests were used to compare PL_ADP_ between groups. The time to primary endpoint between groups was compared using the Kaplan–Meier method. Survival curves were compared using the log-rank test and hazard ratios were calculated using Cox’s regression models. Sensitivity and specificity of PL_ADP_ in predicting thrombotic events were calculated at different thresholds by receiver operating characteristic  (ROC) curve analysis. A two-sided *P* < 0.05 was statistically significant.

## Results

Among the enrolled patients, 1709 completed the 12-month clinical follow-up (Fig. 1). There were 45 (2.6%) ischemic events and 328 (19.2%) bleeding events. Ischemic events included 20 deaths, 20 MI, 21 ST and 11 TVR. Bleeding events included 5 major bleeding, 27 minor bleeding and 296 minimal bleeding.

### Relationship between PR and 1-year outcome

**Fig. 2:**
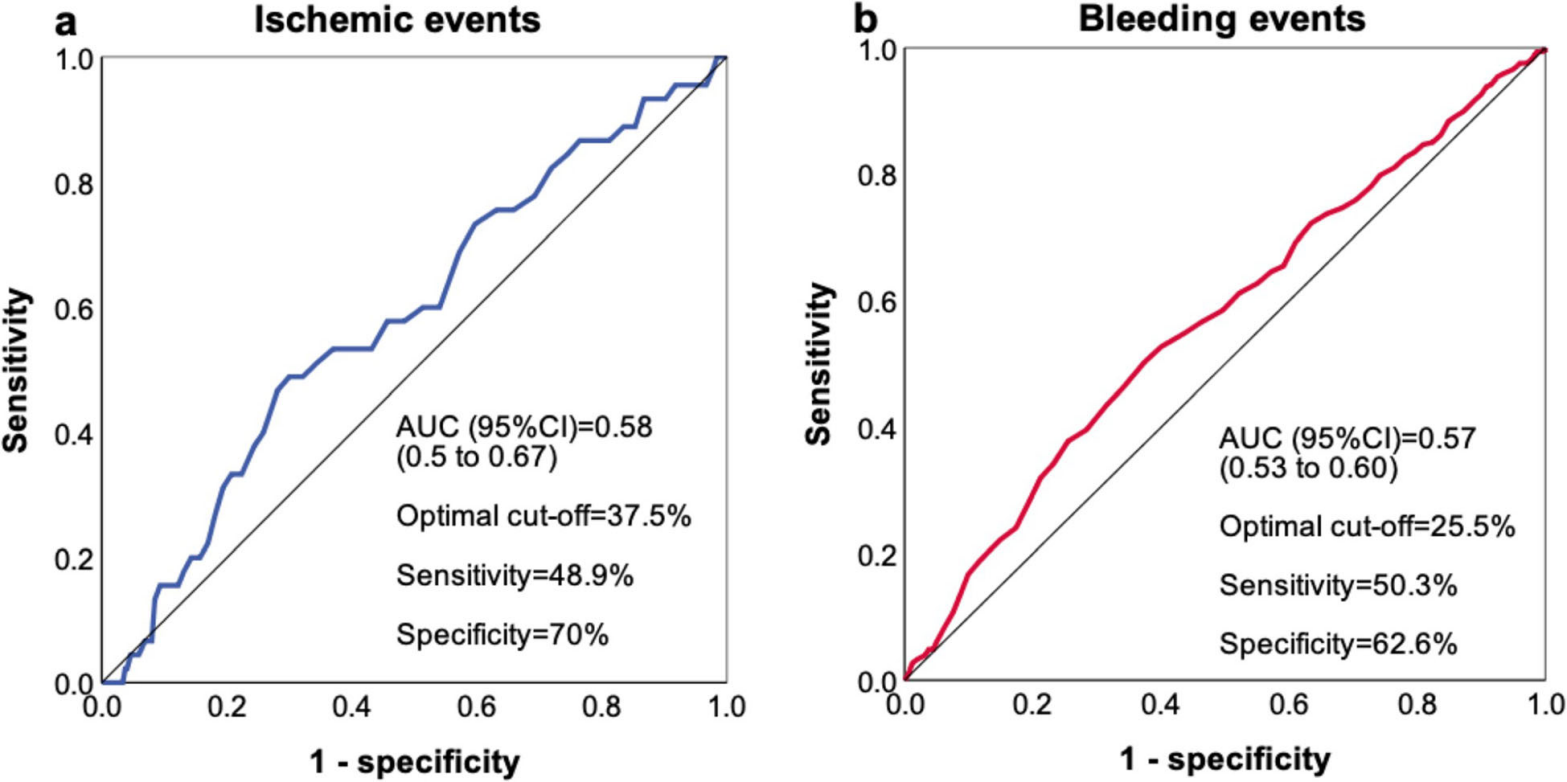
ROC Curves for Ischemic and Bleeding Events. (a) Receiver operating characteristic (ROC) analysis for ischemic events. (b) ROC analysis for bleeding events. AUC, area under the curve; CI, confidence interval; PL_ADP_, ADP induced platelet aggregation

**Fig. 3:**
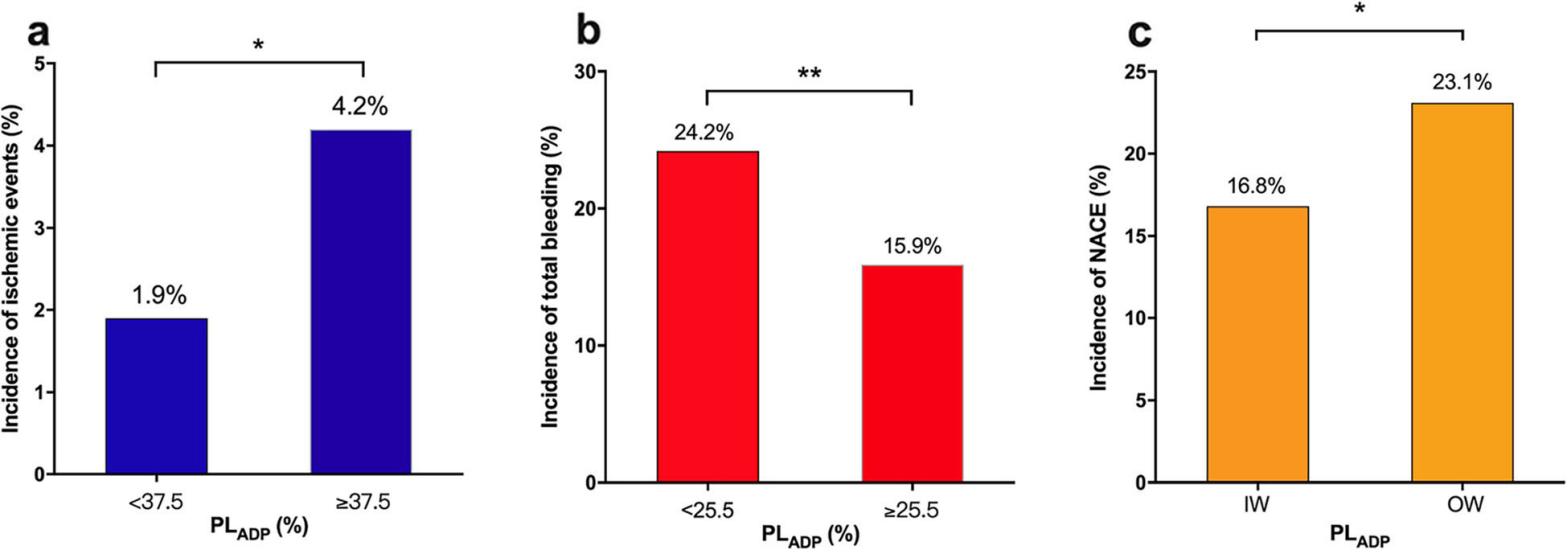
Incidence of Ischemic, Bleeding Events and NACE Stratified by Platelet Reactivity. (a) Incidence of Ischemic events; (b) Incidence of Bleeding events; (c) Incidence of NACE. *P < 0.05; **P < 0.001. PL_ADP_, ADP induced platelet aggregation; NACE, net adverse clinical events; IW, inside the window; OW, outside the window

The average time from PCI to PR test reached 2.50 days. Patients with ischemic events during follow-up had a higher PL_ADP_ level compared to those without (36% [IQR: 25–45] vs.29% [IQR: 20–40]; *P* = 0.054). ROC analysis was performed to evaluate the value of PL_ADP_ in predicting ischemic events. As a result, a PL_ADP_ cut-off value of 37.5% provided a sensitivity of 48.9%, specificity of 70%, and the largest area under the curve value of 0.58 (Fig. 2a). By comparison, the recommended cut-off value of 46% by LTA provides a sensitivity of 20% and a specificity of 84.3% [12]. While adopting 37.5% as a new cut-off value, 521 patients (30.5%) were defined with HOPR, who experienced a higher rate of ischemic events compared with those without (4.2% vs. 1.9%; *P* = 0.007, Fig. 3a).

On the other hand, patients who experienced bleeding events had significantly lower PL_ADP_ compared with those without bleeding (25% [IQR 18–38] vs.30% [IQR 21–41]; *P* < 0.001). By ROC analysis, a cut-off value of 25.5% provided a sensitivity of 50.3%, a specificity of 62.6%, and the largest area under the curve of 0.57 in predicting bleeding (Fig. 2b). Using this new cut-off value, 682 (39.9%) patients were defined with LOPR, who experienced a higher rate of bleeding events compared to those without (24.2% vs. 15.9%; *P* < 0.001, Fig. 3b).

**Fig. 4:**
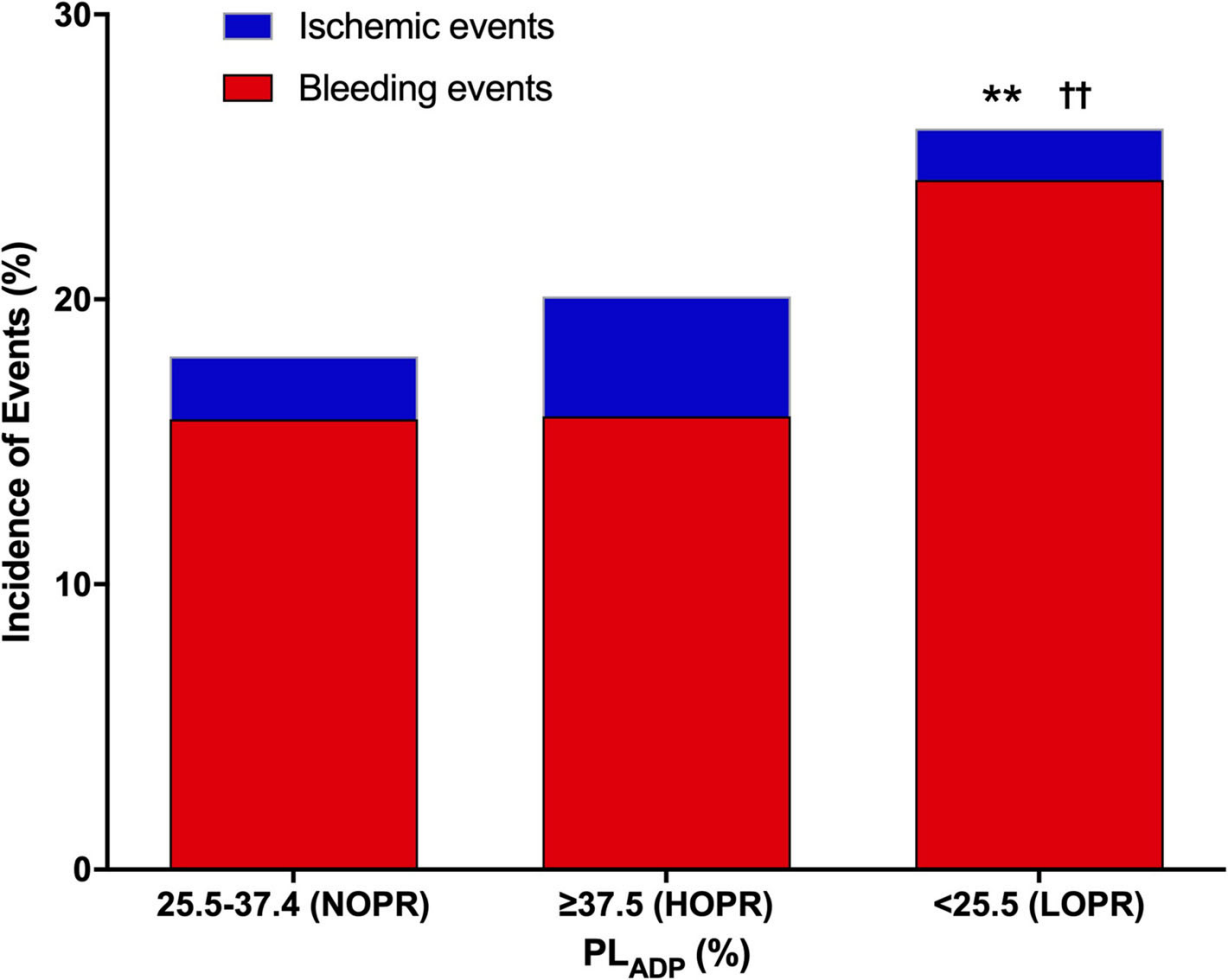
1-year Adverse Events in Groups of Different Level of PL_ADP_. Patients were stratified into groups of NOPR (25.5–37.4%), HOPR (≥37.5%) and LOPR (< 25.5%). ** represents *P* < 0.001 for bleeding events compared with the NOPR group. †† represents *P* < 0.001 for net adverse clinical events compared with the NOPR group. PL_ADP_, ADP induced platelet aggregation; NOPR, normal on-treatment platelet reactivity; HOPR, high on-treatment platelet reactivity; LOPR, low on-treatment platelet reactivity

The risk of ischemic events and NACE was non-significantly higher in patients with HOPR compared with those in normal responders (4.2% vs. 2.2%; HR 1.99; *P* = 0.063 and 19.8% vs. 16.8%; HR 1.19; *P* = 0.247, for ischemic events and NACE, respectively) (Table 1, Fig. 4), while the risk of total bleeding and NACE was significantly higher in patients with LOPR compared with those in normal responders (24.2% vs. 15.8%; HR 1.61; *P* = 0.001 and 25.7% vs. 16.8%; HR 1.64; *P* < 0.001, for bleeding and NACE, respectively) (Table 1, Fig. 4).

**Table 1:** Multivariate analysis based on PL_ADP_ tri-classification

One-year outcome	PL_ADP_						
	Normal responder^*^ n = 506	HOPR^†^ *n* = 521			LOPR^‡^ *n* = 682		
	n(%)	n(%)	HR(95%CI)	P	n(%)	HR(95%CI)	*P*
Net adverse clinical events	85 (16.8)	103 (19.8)	1.19 (0.89,1.61)	0.247	175 (25.7)	1.64 (1.25,2.14)	0.000
Ischemic events	11 (2.2)	22 (4.2)	1.99 (0.96,4.10)	0.063	12 (1.8)	0.83 (0.37,1.89)	0.660
Death	5 (1.0)	12 (2.3)	2.34 (0.82,6.66)	0.111	3 (0.4)	0.45 (0.11,1.88)	0.273
MI	5 (1.0)	9 (1.7)	1.78 (0.60,5.31)	0.303	6 (0.9)	0.93 (0.28,3.05)	0.903
ST	5 (1.0)	11 (2.1)	2.23 (0.77,6.43)	0.138	5 (0.7)	0.78 (0.23,2.70)	0.693
TVR	1 (0.2)	6 (1.2)	5.86 (0.70,48.86)	0.102	4 (0.6)	3.33 (0.37,29.89)	0.282
Bleeding events	80 (15.8)	83 (16.1)	0.99 (0.73,1.36)	0.967	165 (24.2)	1.61 (1.23,2.12)	0.001
Major + Minor	10 (2.0)	5 (1.0)	0.47 (0.16,1.39)	0.172	17 (2.5)	1.17 (0.53,2.60)	0.701
Minimal	70 (13.8)	78 (15.1)	1.09 (0.78,1.52)	0.603	148 (21.7)	1.59 (1.19,2.13)	0.002

Risk factors included in the analysis of net clinical outcome: Sex, age, BMI, Smoking, Hypertension, Diabetes, CABG history, PCI history, Hemoglobin, Platelet count, eGFR, APTT, INR; Risk factors included in the analysis of MACE: Sex, Age, BMI, Smoking, Hypertension, Diabetes, CABG history, PCI history; Risk factors included in the analysis of bleeding: Sex, Age, Hypertension, Diabetes, Hemoglobin, Platelet count, eGFR, INR, APTT

^*^Normal responder: 25.5% ≤ PL_ADP_ < 37.5% (control group); ^†^ HOPR: PL_ADP_ ≥ 37.5%; ^‡^ LOPR: PL_ADP_ < 25.5%

*PL*_*ADP*_ ADP induced platelet aggregation, *HOPR* high on-treatment platelet reactivity, *LOPR* low on-treatment platelet reactivity, *HR* hazard ratio, *CI* confidence interval, *MI* myocardial infarction, *ST* stent thrombosis, *TVR* target vessel revascularization, *BMI* body mass index, *CABG* coronary artery bypass grafting, *PCI* percutaneous coronary intervention, *eGFR* estimated glomerular filtration rate, *APTT* activated partial thromboplastin time, *INR* international normalized ratio

### Optimal PR or therapeutic window of PR to prevent ischemic and bleeding events

According to the ROC curve analysis, we defined an optimal window of PL_ADP_ between 25.5 and 37.5% after dual antiplatelet treatment. As a result, 29.6% of the study population was comprised within this therapeutic window in this study.

We classified the patients into 2 groups according to PR: inside the window group (IW) [PL_ADP_(25.5–37.4%)] and outside the window group (OW) (PL_ADP_ < 25.5% or ≥ 37.5%). The baseline demographic characteristics, clinical, angiographic and biological characteristics and medication history were described in Table 2. There were no significant differences in all the baseline characteristics between the 2 groups.

**Table 2:** Baseline characteristics and medications

	IW*	OW†	*P*
	*n =* 506	*n* = 1203	
Age	65 (58,72)	64 (56,71)	0.104
Gender (%)	74.70	75.40	0.763
BMI (kg/cm^2^)	24.73 (22.84,26.60)	24.51 (22.76,26.54)	0.702
History of CABG (%)	1.20	0.70	0.376
History of PCI (%)	10.50	8.10	0.122
Cardiovascular risk factor			
Smoking (%)	46.20	46.30	0.983
Hypertension (%)	68.00	64.70	0.188
Diabetes (%)	26.50	25.10	0.551
Angiography and intervention			
SYNTAX score	15 (9,21.50)	15 (9,21.50)	0.44
Length of stent	39 (24,66)	39 (24,62)	0.856
Number of stent	2 (1,3)	2 (1,3)	0.737
Biology			
HB(g/L)	136 (125,146)	136 (125,146)	0.988
PLT (×10^9^)	186 (157,227)	186 (155,222)	0.996
LDL (mmol/L)	2.57 (2.06,3.24)	2.49 (2.04,3.12)	0.382
eGFR (ml/min.1.732)	89.51 (77.34,103.95)	89.59 (76.43,104.85)	0.93
APTT (s)	25.90 (23.50,28.40)	25.70 (23.60,28.20)	0.787
INR	1.02 (0.98,1.07)	1.02 (0.98,1.06)	0.609
Medications			
ACEI/ARB (%)	57.70	56.20	0.564
β blocker (%)	66.80	65.10	0.497
Statin (%)	96.40	96.20	0.815
IIb/IIIa inhibitor (%)	2.20	1.20	0.108

Data are presented as median (interquartile range) or percentage as appropriate. * IW: 25.5% ≤ PL_ADP_ < 37.5%; † OW: PL_ADP_ < 25.5% or ≥ 37.5%

*IW* inside the window, *OW* outside the window, *BMI* body mass index, *CABG* coronary artery bypass grafting, *PCI* percutaneous coronary intervention, *MACE* major adverse clinical events, *HB* Hemoglobin, *PLT* platelet count, *LDL* low density lipoprotein, *eGFR* estimated glomerular filtration rate, *APTT* activated partial thromboplastin time, *INR* international normalized ratio, *ACEI/ARB* angiotension converting enzyme inhibitors/ angiotension receptor blocker, *IIb/IIIa* glycoprotein IIb/IIIa inhibitors

**Fig. 5:**
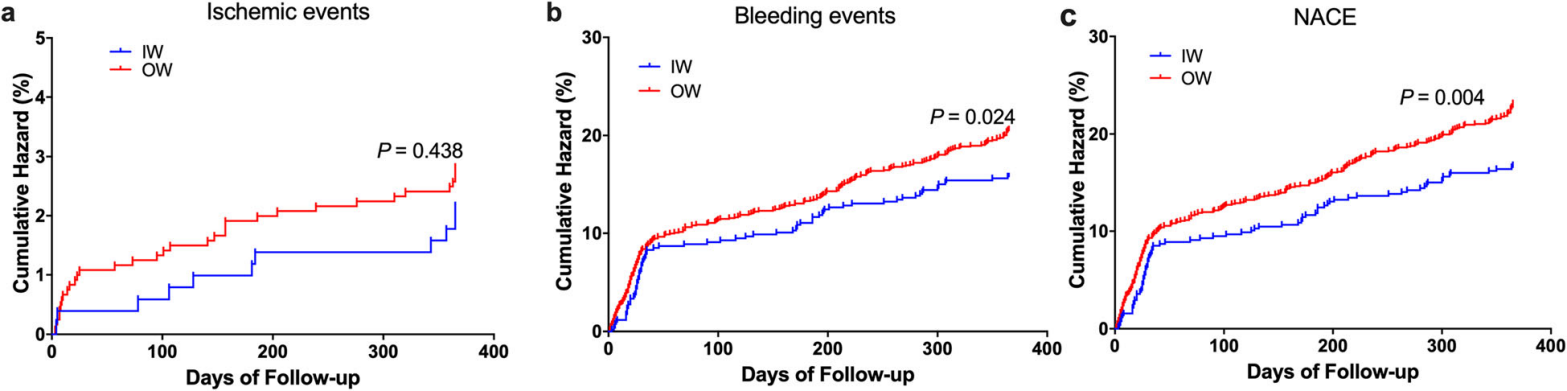
Kaplan-Meier Analysis of Clinical Events. (a) Kaplan-Meier Analysis of Ischemic events; (b) Kaplan-Meier Analysis of Bleeding events; (c) Kaplan-Meier Analysis of NACE. NACE, net adverse clinical events; IW, inside the window; OW, outside the window

We further analyzed the prognosis according to the newly defined therapeutic window. The NACE rate of the IW group patients was lower than that of the OW group patients (16.8% vs. 23.1%; *P* = 0.004) (Fig. 3c). Kaplan-Meier analysis showed a significant difference in NACE and bleeding between patients within and outside the window, although no significant difference was found in ischemic events (*P* = 0.438, 0.024 and 0.004, for ischemic events, bleeding and NACE, respectively)(Fig. 5). The hazard ratio of NACE for OW group was significantly higher during the 12-month follow-up compared with IW group [1.44 (95% CI: 1.12–1.85; *P =* 0.004)] after adjusting for age, gender, body mass index (BMI), history of smoking, hypertension, diabetes, coronary artery bypass grafting (CABG), PCI, hemoglobin, platelet count, estimated glomerular filtration rate (eGFR), activated partial thromboplastin time (APTT), and international normalized ratio (INR) (Table 3). The total bleeding rate was also significantly higher in OW than IW after adjusting for the confounders [1.33 (95% CI: 1.03–1.72; *P* = 0.028)], which turned out to be the main contributor to NACE (Table 3).

**Table 3:** Multivariate analysis based on the therapeutic window

One-year outcome	IW^*^	OW^†^	HR	95%CI	*P*
	506	1203			
	n(%)	n(%)			
Net adverse clinical events	85 (16.8)	278 (23.1)	1.44	1.12–1.85	0.004
Ischemic events	11 (2.2)	34 (2.8)	1.34	0.68–2.64	0.403
Death	5 (1.0)	15 (1.2)	1.27	0.46–3.51	0.640
MI	5 (1.0)	15 (1.2)	1.30	0.47–3.59	0.608
ST	5 (1.0)	16 (1.3)	1.41	0.52–3.87	0.500
TVR	1 (0.2)	10 (0.8)	4.49	0.57–35.17	0.150
Bleeding events	80 (15.8)	248 (20.6)	1.33	1.03–1.72	0.028
Major + Minor	10 (2.0)	221 (1.8)	0.86	0.40–1.84	0.700
Minimal	70 (13.8)	226 (18.8)	1.38	1.05–1.81	0.022

Risk factors included in the analysis of net clinical outcome: Sex, age, BMI, Smoking, Hypertension, Diabetes, CABG history, PCI history, Hemoglobin, Platelet count, eGFR, APTT, INR; Risk factors included in the analysis of MACE: Sex, Age, BMI, Smoking, Hypertension, Diabetes, CABG history, PCI history; Risk factors included in the analysis of bleeding: Sex, Age, Hypertension, Diabetes, Hemoglobin, Platelet count, eGFR, INR, APTT

^*^ IW: 25.5% ≤ PL_ADP_ < 37.5%; ^†^ OW: PL_ADP_ < 25.5% or ≥ 37.5%

## Discussion

In this study, we identified an optimal range of platelet reactivity as 25.5–37.4% for PL_ADP_ while determined by LTA for patients underwent PCI and on the treatment of regular-dose aspirin and clopidogrel, and approximately one third (29.6%) of the patients meet this therapeutic window. Patients inside the window presented significantly lower risk of NACE than those outside the window during 12-month follow-up.

Several studies have tried to identify a threshold of PR that could stratify patients at risk of ischemic events. Bliden et al. [17] found that HOPR (defined as PL_ADP_ ≥ 50% measured by LTA with ADP concentration of 5 μmol/L) was the only variable being significantly related to ischemic events after adjusting for hypertension, diabetes and use of calcium channel inhibitors. Gurbel et al. [6] demonstrated that HOPR (defined as PL_ADP_ ≥ 46% measured by LTA [12] with ADP concentration of 5 μmol/L) was an independent risk factor for ischemic events within 2 years of non-emergent PCI (OR = 3.9, *P*< 0.001).

The cut-off value of PL_ADP_ in our study is 37.5%, which is lower than the previous study. However, as demonstrated by the GRAVITAS trial, when HOPR was defined as ≥230 P2Y_12_ reaction units (PRU) by VerifyNow P2Y_12_ test, high-dose clopidogrel compared with standard-dose clopidogrel did not reduce the incidence of major adverse cardiovascular events [18], while the post-hoc analysis found that the achievement of a PRU < 208 was associated with significantly improved clinical outcomes. Consistent with the GRAVITAS trial, our result suggests that a lower cut-off value of PL_ADP_ might bring more low responders to the intensified anti-platelet treatment and consequently reduce ischemic events.

In addition to recurrent ischemic events, the prognostic importance of bleeding complications following PCI has also been established. ADAPT-DES trial showed that HOPR (defined by > 208 PRU, by VerifyNow P2Y_12_ test) was inversely related to TIMI major bleeding (adjusted HR: 0.73, 95% CI: 0.61 to 0.89, *P* = 0.002) [3]. Studies suggested a possible link between LOPR and bleeding [7–9, 18–23]. With the LTA method, Tsukahara et al. [24] found that high-responsiveness was the independent predictor of major bleeding in patients receiving drug-eluting stents and treated with thienopyridine. Parodi et al. [25] reported that LOPR (PL_ADP_ < 40%, 10 μmol/L ADP, LTA assay) were the independent predictor of bleeding events. Consistent with previous studies, we confirmed the predictive value of PR on the occurrence of bleeding events after PCI as measured with the LTA assay, and we suggested a cut-off value of PL_ADP_ < 25.5% to predict the bleeding events.

The optimal therapeutic window of PL_ADP_ is uncertain, Campo [26] and Mangiacapra et al. [1] have reported two therapeutic windows for PR measured with the VerifyNow P2Y_12_ assay. However, in Campo’s study, they reported all clinical events (ischemic and bleeding) after 1 month and up to 1 year of follow-up. Patients with adverse events during the first month were excluded. In Mangiacapra’s study, only short-term outcome of 1-month clinical events were analyzed. By contrast, using the two thresholds for ischemic and bleeding events, we found an optimal therapeutic window for PL_ADP_ by LTA assay, ranging from 25.5 to 37.4%, which was associated with the lowest 1-year incidence of NACE. To the best of our knowledge, our study was the first that use LTA method to demonstrate an optimal therapeutic window for PL_ADP_ regarding the 1-year clinical outcome.

Our study has important clinical implications. According to the results, post-PCI evaluation of PR carries important prognostic information, and the antiplatelet treatment should be guided referring to optimal therapeutic window of PR instead of single cut-off value. In particular, for patients with HOPR and higher ischemic risk, more aggressive antiplatelet strategies might be useful. On the other hand, for patients with LOPR and higher bleeding risk, conservative antiplatelet therapies should also be indicated until PR falls within the desired range.

The present study has potential limitations. First, the limited funding support prevented us to perform another cohort to validate the study results. Thus, a prospective study would be needed before using such an assay to try to predict outcomes. Second, the sample size was modest, so we could not analyze the optimal ranges of platelet reactivity for different age groups. Third, platelet reactivity could vary while patients taking clopidogrel treatment for longer term. However, we could not further extend the time of platelet reactivity test due to the limited hospitalization period. Besides, patients would be on high risk of thrombotic events early after PCI, so clopidogrel response in early stage of stent implantation would be more important to overcome or predict the thrombotic events.

## Conclusion

An optimal therapeutic window of 25.5–37.4% for PL_ADP_ predicts the lowest risk of net adverse cardiovascular events, which could be referred for tailored antiplatelet treatment while using platelet aggregation assay by light transmittance aggregometry.

## Data Availability

The datasets used or analyzed during the current study are available from the corresponding author on reasonable request.
